# Education and training of clinical oncologists—experience from a low-resource setting in Zimbabwe

**DOI:** 10.3332/ecancer.2021.1208

**Published:** 2021-03-23

**Authors:** Ntokozo Ndlovu, Sandra Ndarukwa, Albert Nyamhunga, Patience Musiwa-Mba, Anna Mary Nyakabau, Webster Kadzatsa, Melinda Mushonga

**Affiliations:** 1 University of Zimbabwe Faculty of Medicine and Health Sciences, Harare, Zimbabwe; 2 Parirenyatwa Hospital Radiotherapy Centre, Harare, Zimbabwe; 3 Sally Mugabe Central Hospital, Harare, Zimbabwe

**Keywords:** training, radiotherapy, oncology, curriculum, collaboration, Zimbabwe

## Abstract

As the burden of cancer increases worldwide, more so in low- and middle-income countries, one of the greatest challenges is human resource capacity development. Addressing this is critical in reducing the burden of cancer in the African continent. Other challenges include socio-economic demographics and disparities in the overall cancer care. Lack of sufficient numbers of qualified staff has been one of the obstacles in developing adequate and modern cancer treatment centres in Africa. Training in clinical oncology in Zimbabwe was established in 1990 through the collaboration between the Government of Zimbabwe and the WHO as a regional project. The training is offered by the University of Zimbabwe through the established Master of Medicine in Radiotherapy and Oncology (MMed Rad & Onco) postgraduate programme. Regional and local fellows have been trained, yielding more than 20 clinical oncologists over the years, who have initiated cancer treatment facilities in Africa and beyond. They have continued to train others, fulfilling the original WHO programme target of transfer of skills in sub-Saharan Africa. Collaborations with external partners have complemented efforts by the local faculty in addressing deficiencies in training, in areas where experts in the subject are lacking and in supporting nationals working abroad to come and teach newer technologies and techniques. The curriculum continues to evolve from knowledge-based training to competency-based training. However, there is a need to expand the current infrastructure to keep up with changing technology.

Clinical oncology training in Zimbabwe continues and remains a regional resource. Emphasis on subspecialising seems to be the next natural step in progression. Strengthening of other disciplines, including surgical oncology and medical physics, would be complementary to the training. The programme is an example of a sustainable initiative born out of collaborative partnership and is sustained by local resources. The greater majority of qualified oncologists have remained in Africa.

## Introduction

The burden of cancer is increasing worldwide and more so in Africa. One of the challenges faced in addressing the impending cancer epidemic is human resource capacity development. The oncology workforce is well known to be depleted globally [1]. This is more pronounced in low- and middle-income countries (LMICs) due to a number of factors in healthcare delivery systems. One of them is limited prioritisation of healthcare (in particular cancer care) in such countries [2, 3]. In Africa, human resource deficiency in cancer care is more marked due to the socio-economic demographics and enormous disparities in radiotherapy service provision. About half of the continent has no access to radiotherapy facilities at all [4, 5]. Lack of personnel is more pronounced in radiation than medical oncology with subspecialising being a rarity. Education and training in radiotherapy is thus of utmost importance as it is essential for the provision of quality and adequate service delivery [6].

There is very little documentation of oncology training programmes in Africa, particularly those involving radiotherapy, such as clinical or radiation oncology (RO). Radiotherapy treatment is highly technical in nature and services need to be adequately staffed with well-trained personnel to ensure accuracy and key safety elements in treating cancer patients. In an International Atomic Energy Agency (IAEA) survey conducted in the African region in 2013, the lack of qualified staff in sufficient numbers was stated to be one of the main obstacles to the development of adequate and modern radiotherapy centres in Africa. The study which was conducted in the framework of a Regional Project RAF6045 assessed the existence, number and location of education programmes for the five main radiotherapy professions: RO, medical physics (MP), radiation therapy technician (RTT), radiation biology and oncology nursing. In the findings, there were 39 education programmes in radiation oncology, 36 in MP, 45 for RTTs, 14 in radiation biology and 21 for oncology nurses. Nine countries were following the clinical oncology model. A review of the minimal standards of quality of the programmes could not be undertaken in this particular survey [7].

Similarly, a survey on the global cancer workforce that was published as a perspective of the African cancer workforce highlighted the lack of investment in healthcare professionals who deliver cancer care. Oncologists in Africa were shown to be overburdened with workloads well above those of their peers elsewhere. They also were of a higher average age and were mainly medical oncologists [8]; considering that radiation/clinical oncologists are even fewer in Africa, this suggests a greater need for training and support of oncologists to be able to deliver radiotherapy.

In yet another global survey of the clinical oncology workforce it was recommended that training programmes be instituted in regions with an extreme shortage of clinical oncologists, with governments addressing the challenges within their regions. The training of doctors and nurses from low-income countries, by sending them to high-income countries, was discouraged in favour of the available funds being used to enhance regional collaborations [9].

This manuscript describes the clinical oncology training programme in Zimbabwe and its progression, whose inception was previously reported by Stjernsward *et al* [10]. It is one of the postgraduate clinical programmes offered by the University of Zimbabwe, Faculty of Medicine and Health Sciences. Trainees graduate with a Master of Medicine in Radiotherapy and Oncology (MMed Rad & Onco) after 4 years of training. They then need to practice under supervision for 1 year to register as specialists in Clinical Oncology.

## The need to train oncologists

The incidence of cancer is rising in Zimbabwe. In the year of inception of the oncology training programme in Zimbabwe, a total of 4,659 cancers were recorded by the Zimbabwe National Cancer Registry (ZNCR). This number has steadily increased to over 7,000 new cancer cases being recorded annually.

**Figure 1.**
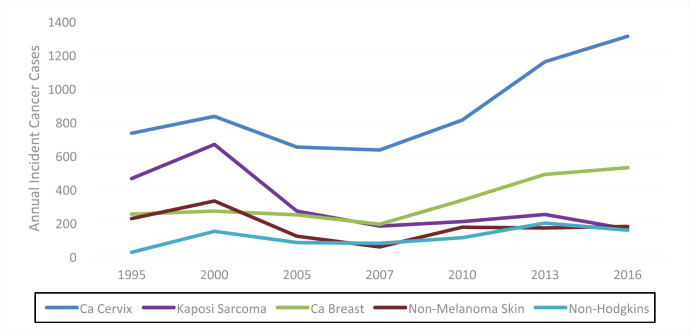
Trends of female cancers in Zimbabwe from 1995 to 2016.

Figure 1 shows the cancer trends of the five commonest cancers in Zimbabwean women over the period of 1995–2016, with verified reports available. Clearly, the increase in the incidence of cervical and breast cancers is the main driver of this general increase in cancers in women against a decrease in Kaposi sarcoma that signals the control of the HIV epidemic. The 2017–2019 unpublished data also show a similar upward trend.

**Figure 2.**
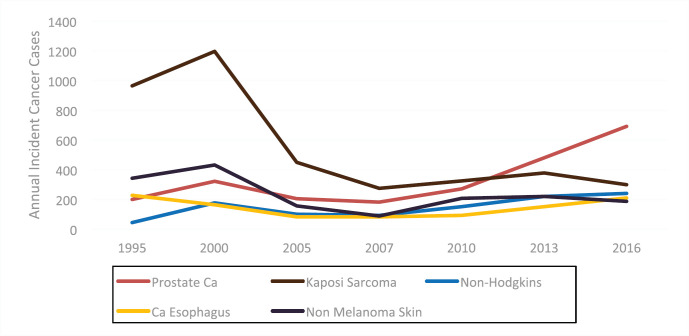
Trends of male cancers in Zimbabwe from 1995 to 2016.

In the male population during the same period, the main driver of increased cancer incidence was prostate cancer. A large decrease in Kaposi sarcoma incidence was also noted in men as in women (see Figure 2).

The latest edition of the NCRZ (2016) also showed that 87% of the cases presented with TNM stages 3 and 4 (10). In view of the above trends in the incidence and advanced stages of the disease at presentation, radiation is an important component of cancer care in Zimbabwe as is in most LMICs and globally. To address the treatment needs, there are three radiotherapy centres in the country, of which two are public centres. Two centres are located in the capital, Harare, one of these being a private centre with one linear accelerator and the other being the centre affiliated to the Academic Hospital (with three linear accelerators). The third centre is situated in the second largest city, Bulawayo, and is also an academic centre for the National University of Science and Technology and UZ. It is a public centre with two linear accelerators and services the southern part of the country.

## Inception of oncology training

The training of clinical oncologists was set up in Zimbabwe in 1990 to address the growing cancer burden against the depletion of human resources and in response to the huge gap that had been observed in cancer management in the sub-Saharan region. It was with full recognition that such a development could not be done in isolation from other cancer control activities. Appropriate priorities and strategies were systematically put in place so that efforts towards cancer treatment could be linked to other comprehensive cancer control efforts. This would include prevention, early detection and palliative care for cancer patients, all being given importance. The WHO, in collaboration with the Government of Zimbabwe, initiated a national and regional training programme in clinical oncology under the auspices of the UZ. This programme was initiated with a suitably adapted syllabus aimed at addressing the then set priorities and strategies that would build into the national cancer control programme [10, 11].

Fellows from Zimbabwe and the African region were then enrolled to be trained at the Parirenyatwa Hospital Radiotherapy Centre and continue to be trained in this programme. Three WHO consultants from different backgrounds and professional strengths facilitated the training. The consultants were two clinical oncologists from the United Kingdom and one radiation oncologist from the United States of America. They were complemented by one local clinical oncologist and two local medical physicists. The choice of location of Zimbabwe was influenced by the availability of infrastructure and the local potential in other disciplines that existed to support the training and contribution to the multidisciplinary teams. The programme culminated in the training of clinical oncologists from a number of African countries. Critical cancer treatment capacity in the host country was also strengthened as the number of trained oncologists increased (see Table 1). The management of the programme was transferred to the local university in 1995 and subsequently continues with local staff who are fully responsible for its running. Following the training of the first eight oncologists by the WHO experts, more than 20 clinical oncologists have graduated from this collaborative programme. Many have initiated cancer treatment facilities in Africa and others have contributed to the field of Oncology beyond the continent. The programme continues to run under the full purview of the UZ and is funded like all other university degree programmes with trainees usually employed by the teaching hospital and paying tuition fees to the UZ.

## Staffing and collaborative partnerships in training

Following the completion of training by the first and subsequent cohorts, clinicians from the programme have continued to give back by training other oncologists. This has been the case in Zimbabwe and some of the countries where the trainees originated, fulfilling the original plan of the WHO project that targeted the transfer of oncology skills within the continent of Africa [10].

**Figure 3.**
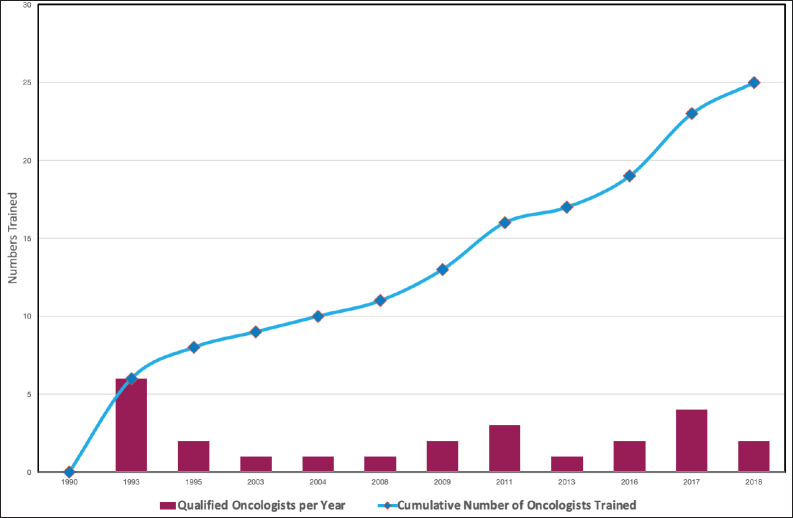
Trends in the number of qualifying oncologists.

Such training must output highly profiled specialists who are able to make sound independent decisions in various scenarios and settings. This requires certain conditions to be met in training, including adequate mentorship through adequate staffing levels, to ensure accuracy in the delivery of radiotherapy [12]. This influenced the choice of the three WHO experts from different backgrounds who were tasked with the setting up of the training programme. Initial intake was six trainees from four countries. The trainee–mentor ratio was as recommended for other similar training programmes worldwide as it ensures sufficient contact hours and adequate mentoring [13]. Since then, the aim has been to maintain this equilibrium. Even though the need to train more oncologists has been a pressing issue, the size of intake has always been a limited number of trainers at any given time (see Figure 3).

The current academic department is able to support an intake of four to six trainees per year. There is provision for four full-time teaching staff for the programme. Part-time staff are recruited according to the need to cover any gaps in teaching. Currently, the local faculty is composed of two full-time and four part-time clinical oncologists and one part-time medical physicist. Only two members of the staff are male. There is a provision for visiting lecturers from other institutions worldwide and similar reciprocal visits by local faculty. Continuous medical education for staff is key, with a scheme that is monitored by professional regulatory bodies on an annual basis and being conditional to licence to practice.

Economic challenges have led to brain drain, periodically resulting in slowing down the growth of the faculty. The ultimate goal, which is to obtain a critical mass of excellence and knowledge locally, so that Zimbabwe can serve as a future centre for the training of African doctors by African doctors in cancer control without the need for extensive training abroad has, however, been achieved.

With a small faculty, such as in this institution, innovation is needed to address areas of potential deficiency. One such identified area is the teaching of radiobiology which is a challenge to most sub-Saharan training programmes in oncology and beyond [14, 15]. For this programme, the challenge has been resolved through a block release approach that involves outsourcing expertise to deliver the module in a short period of time. Collaboration with the IAEA through the involvement in regional courses and from scientific visits complements efforts by the local faculty. Innovative strategies to teach radiobiology have been implemented elsewhere in Africa, using the example of the Zimbabwean experience. Also, wherever possible interfaculty or interdepartmental teaching is instituted, examples being teaching cancer pathology/biology, palliative care and medical statistics. Furthermore, the International Organisation of Migration supports nationals who emigrated to greener pastures with prerequisite professional skills, to temporarily return and impart knowledge on newer technologies and techniques in radiotherapy as experienced in countries with more advanced equipment.

Many other collaborative efforts have borne fruit in filling the gaps and upgrading the training programme, one being the multidisciplinary cancer management course that was designed by the American Society of Clinical Oncology staff and local experts to provide a roadmap for cross-specialty interaction and coordination of care in Zimbabwe. The cancer management course was relevant to daily practice, fostered long-lasting partnerships and collaborations, and resulted in a more motivated local workforce. It also strengthened the existing multidisciplinary practices as a cost-effective strategy with sustainable benefits [16].

## The curriculum and assessments

At the inception of the programme, it was important to design a syllabus that would be relevant to the individual country’s cancer control situation without compromising on quality; this was considered to be essential [10]. The course content was modelled against the United Kingdom (UK) Clinical Oncology syllabus which included teaching mainly focused on developing knowledge (subject and time-based) and this has remained the backbone of clinical oncology training in Zimbabwe.

For entry into the programme, applicants should have first obtained a medical degree of this or another university of an approved standard or have obtained a qualification by written and clinical examination of a standard approved by the University of Zimbabwe. Applicants must have completed a suitable internship and be appropriately registered with the Medical and Dental Practitioners Council of Zimbabwe. The curriculum comprises two phases of training as summarised in Table 2. The first phase is training in basic sciences. The second phase is training the candidate to have an excellent knowledge of managing all aspects of cancer with an emphasis on both radiotherapy and chemotherapy and having possession of all the required competencies.

The course content has been regularly updated as treatment methods and radiotherapy techniques evolve. The existing syllabus has been recently reviewed to include applied anatomy and physiology as subjects in the first phase of training and translational sciences as a subject in the second phase of training. This form of training is still standard in most countries, but with the introduction of competency-based training around 2017 some institutions, especially in North America and Europe, have since changed their curricula [17, 18]

With the major changes occurring in medical education, including the embracing of competency-based teaching methods, the Zimbabwean Oncology curriculum is being reviewed to reflect this approach. This has come from a realisation that delivering high-quality health services requires a more complex approach that fosters training of skills with an outcome-based approach using an organised framework of competencies. This includes skills in clinical decision-making, communication skills along with knowledge and attitude required to apply those skills appropriately as in the Canadian Medical Education Directives for Specialists (CanMEDS) framework that identifies and describes the abilities physicians require to effectively meet the healthcare needs of the people they serve [19]. This approach also moves from the traditional instructor or lecturer-driven process to a more learn-by-doing approach focussed on developing specific competencies required, with trainers mostly taking the role of mentors/coaches [6].

The UZ Faculty of Medicine and Health Sciences has advocated for the adoption of the competency-based teaching approach with sensitisation and training offered to all faculties. The Department of Oncology, with the approval of the Department of Health Professionals Education, is in the process of developing the revised curriculum in line with the competencies required for clinical oncology training. A generic competency-based clinical assessment tool was introduced by the UZ in 2018. This assesses students across competencies which include medical knowledge, communication skills, ethical professionalism, leadership and ability to educate others. This assessment is conducted quarterly after each clinical rotation and constitutes 50% of the continuous assessment mark. The development of student portfolios and logbooks was well underway and was expected to be in use by the end of 2020.

Members of the faculty are also involved in the development of the African Regional Cooperative Agreement for Research, Development and Training related to Nuclear Science and Technology (AFRA) curriculum on training clinical oncology for the region. This is in conjunction with the IAEA which has also facilitated an evaluation of the UZ programme by external international experts. This will hopefully contribute to efforts of the development of the departmental curriculum. The oncology curriculum continues to change considerably. These changes, although a challenge to implement, are a necessary component of oncology education. They ensure that trainees are equipped with relevant up-to-date and comprehensive knowledge for the benefit of cancer patients and survivors.

## Cancer treatment infrastructure in the context of education

In 1990, when the programme was initiated, the radiotherapy equipment available was in the form of one linear accelerator (6MV), two cobalt 60, two 250-KV external beam machines, two strontium 90 eye applicators, Caesium manual after loading brachytherapy sources and applicators and two treatment simulators [10]. In that time period, linear accelerator technology was rare in sub-Saharan Africa as was brachytherapy capability. The introduction of a computerised radiotherapy treatment planning (RTPS) system took place in the second year of the programme’s inception. Medium dose remote after loading brachytherapy utilising caesium sources was also procured. The last cobalt teletherapy unit was decommissioned in 2011, making room for the expansion of linear accelerator technology. Currently, the department has three linear accelerators (two photon and four electron energies) treating an average of 1,500 news patients per year, two high-dose remote after loading cobalt brachytherapy machines, an oncology information system and RTPS capable of supporting newer treatment techniques.

The expansion of the infrastructure is very necessary to keep up with the changing technology so as to equip trainees with knowledge that can be applicable in different settings. It is, however, impossible in a low-resource setting to have all the latest technology under one roof. This calls for the utilisation of resources apart from those available in the training institution through student exchange programmes, private–public partnerships and visiting lectureship as they are invaluable in impacting the needed competencies. The cost of new equipment can be prohibitive, but in this case, advocacy and support of the training programme at a high level have ensured that funding is continually made available to sustain the required equipment needs, hence sustaining the training. Equipment maintenance is one area that remains a challenge and is being constantly addressed through engagement with vendors and local administrative structures.

Infrastructure for systemic therapy is less problematic as it requires less financial outlay compared to radiotherapy. The curriculum is designed to ensure that students are well trained to deliver the state-of-the-art therapies of established efficacy for the common cancers within the framework of care for individual patients. Concerns are more on the availability of drugs as most novel therapies are very costly and may not be marketed in low-resource settings.

The medical oncology training within this programme is designed to deliver core specialty training by the acquisition of knowledge and skills as assessed by the workplace-based assessments. The clinical modules are completed randomly as there are no specific clinics for diseases’ sites and, therefore, students are assessed based on the cases managed over the whole period of the training. This makes room for periodic shortages of drugs and the use of private facilities to provide a wider clinical experience.

## Research

Research is an important and integral component in education and training, and the curriculum, therefore, stresses on its importance. There is a module that requires candidates to conduct a supervised research project in partial fulfilment of the programme’s requirements. In preparation for these projects, the candidates participate in different modules including medical statistics and epidemiology which are taught subjects in the first year of the programme. The research projects allow the students to be exposed to the real-world expectations in the research field, from formulating research questions and hypotheses, study designs, ethical considerations, data collection and management, data analysis and dissemination of the research findings. Several research projects from this programme have been presented at different international forums, winning awards and scholarships for the candidates to further their research careers. Some of these projects are published in peer-reviewed journals and contribute to the international data.

Earlier in the first few years of the programme’s inception, efforts were made towards initiating multidisciplinary research involving the oncology faculty. Some of the research activities then resulted in practice-changing findings such as in the management of Kaposi’s sarcoma and palliative care [20, 21]. Since that time, there has been growing involvement through IAEA coordinated research projects and other initiatives like the AIDS Malignancy Consortium. This has resulted in participation in international multicentre clinical trials and involvement in several studies. Trainees have benefited from this by being involved at different levels, hence boosting their understanding of the conduct of research. The enhancement of an interdisciplinary oncology approach to research is still needed to improve oncology researcher’s knowledge and skills to participate in research involving basic sciences like immunology and microbiology. Some of the graduates have had the opportunity to be attached to internationally acclaimed centres, through different collaborations, for post-graduate training to reinforce their research skills. Some of the studies conducted by faculty and trainees, as stated above, have improved contribution to local evidence, as well as boosting the confidence of the trainees to generate and make use of the same.

## The future of oncology training in Zimbabwe

Training of clinical oncologists in low-resource settings has some advantages as it produces a cadre who can function in the whole spectrum of oncology practice and is likely to remain in the region, thus reducing the flight of skills. While such a model of combined radiation and medical oncology training is ideal as a starting point where there is no existing training, recognition of the fast development of the field in terms of technologies and newer therapies may necessitate the separation of medical and radiation oncology, with subspecialising. Subspecialising is the next step that is proposed in Zimbabwe. This will be complemented by encouraging the strengthening of other oncology disciplines, such as surgical, gynaecologic, paediatric oncology and MP. Expansion to encompass genetic counsellors, onco-pharmacology training and other related areas will help strengthen multidisciplinary team management, which is the backbone of best-practice oncology.

Standardisation of the curriculum in the African continent will help lessen the impact of limited human resources as online web-based training and common lectures can be taught by a few in the whole continent. Efforts have already started in that direction, driven by a number of regional/international developmental and educational partners, such as the African Organisation for Research and Training in Cancer and IAEA.

The estimated population in Zimbabwe as of 2020 was 14.8 million and 18 of the oncologists trained so far are Zimbabweans. Of these, 14 are currently in practice in the country, with three working in other countries and one having left medical practice. This gives a ratio of one oncologist per 1.057 million population. This is still a long way from the USA figures of one oncologist per 26,418 population, but is comparative to one oncologist per 2 million population within regions such as Kenya. The current programme is expanding well to meet the needs of Zimbabwe. However, the expansion must not be isolated from infrastructural resources and the training of all other multidisciplinary players in cancer care. A national plan is needed towards that.

Public centres offer treatment for free or at very subsidised rates. Most persons in formal employment have medical insurance which covers radiotherapy treatments to varying extents. This is considered to ensure that no one is denied access to radiotherapy needlessly.

## The impact of the global COVID-19 pandemic on training programmes

In the current era of the COVID-19 pandemic, strategies of training and education at our institution have been reviewed to consider the local and global changes that have since occurred. With the adoption of e-learning comes a number of challenges that have not yet been fully explored, such as limited availability of Internet off campus. This is a great challenge in LMICs in general. E-learning in oncology is not new and has been shown to be useful in some settings [22]. In our experience, the challenges are mainly those of clinical training and patient contact which have been reduced. Internet access is limited especially off campus where there are high costs to access the Internet. While only a few patients with cancer have been diagnosed with the infection, local COVID-19 guidelines have been put in place to minimise the risk of infection to patients and staff.

In some countries, academic institutions have recorded huge financial losses due to the loss of income generated through student accommodation, catering and offering conferencing facilities. Upscaling of e-learning facilities due to the pandemic has also increased expenditure. Students are also deferring enrolment. Questions are being asked such as if such downgrading of the student experience is equivalent to a downgrading of the value of the degree and how to deal with students who have missed months of their clinical experience. Only time will tell what changes this pandemic will make to the academic sector [23, 24].

## Conclusion

This article presents one of the clinical oncology training programmes in Africa that has provided the much-needed human resources for sustainable comprehensive oncology programmes. Aligning the programmes to cater for the dynamic nature of oncology needs to be tailor-made to specific country needs.

Countries in the region have many nationals who are qualified in providing clinical oncology services, including radiotherapy. The majority remain abroad after training, with only a few returning to serve their own people. This programme has trained professionals who have remained in the continent and have significantly contributed to the expansion of services in their respective countries of practices. This programme is an example of a sustainable initiative born out of collaborative partnership that has continued to function by being sustained by local resources.

There are many challenges encountered in running such a programme from limited resources. Innovative and dynamic solutions need to be found and implemented. Evolution of training with time to consider changes in technology and new therapies is an important determinant of sustainability

## Conflicts of interest

All authors have no conflicts of interest to declare.

## Source of funding

This work is not funded by any source.

## Figures and Tables

**Table 1.** Oncologists trained by year of graduation, country of origin and country of practice.

Year of graduation	Number graduating (*n* = 25)	Country/countries of origin (*n*)	Country/countries of practice
1993	6	Zimbabwe (3) Cameroon (1) Nigeria (1) Tanzania (1)	Zimbabwe Botswana Cameroon Nigeria Namibia
1995	2	Zimbabwe (1) Zambia (1)	Zimbabwe Zambia
2003	1	Zimbabwe (1)	Zimbabwe Bahamas Botswana
2004	1	Tanzania (1)	Tanzania
2008	1	Zimbabwe (1)	Zimbabwe
2009	2	Zimbabwe (2)	Zimbabwe
2011	3	Zimbabwe (3)	Zimbabwe United Kingdom
2013	1	Zimbabwe (1)	Zimbabwe
2016	2	Zimbabwe (2)	Zimbabwe
2017	4	Zimbabwe (3) Kenya (1)	Zimbabwe Kenya
2018	2	Zimbabwe (1) Kenya (1)	Zimbabwe Kenya

**Table 2.** The Zimbabwean training structure in radiotherapy and oncology.

Phase	Duration	Modules covered
1st phase	1 year and 3 months	Basic Sciences comprising Physics, Pathology, Radiobiology, Anatomy Physiology, Medical statistics and Epidemiology
2nd phase	Remainder of the training period totalling 4 years	Principles of management of cancer patients, site-specific management of cancers Site-specific carcinogenesis, epidemiology and clinical aspects Radiotherapy Chemotherapy (and other systemic therapies) Application of novel treatments Palliative and supportive care Cancer prevention and community-centred care Research work/ dissertation
